# Generation of Simulated “Natural” Nanoplastics from Polypropylene Food Packaging as the Experimental Standard

**DOI:** 10.3390/molecules28217254

**Published:** 2023-10-25

**Authors:** Zhongtang Wang, Ying Wang, Xin Lu, Hongyan Zhang, Zhenzhen Jia

**Affiliations:** Shandong Provincial Key Laboratory of Animal Resistance Biology, Key Laboratory of Food Nutrition and Safety of Shandong Normal University, College of Life Sciences, Shandong Normal University, Jinan 250014, Chinazhanghongyan@sdnu.edu.cn (H.Z.)

**Keywords:** nanoplastic, standard substance, fractional filtration, natural condition simulation

## Abstract

Current toxicology research on nanoplastics (NPs) generally uses commercial spherical NPs. However, the physicochemical characteristics of commercial NPs are significantly different from those of NPs formed under natural conditions, possibly affecting the validity of the results. In analytical chemistry, a reference sample is selected such that its physicochemical properties are as similar as possible to the target. Therefore, a simulated “natural” NP synthesized in the laboratory that closely resembles naturally derived NPs would be used as an authentic standard. Here, we established the assay of scanning electron microscope (SEM)-particle size distribution analyzer (PSDA)-surface-enhanced Raman scattering (SERS) to detect NPs and prepared simulated “natural” NPs from polypropylene food packaging material using a method that mimics natural conditions. Nanofiltration was used to isolate three sets of simulated NPs with particle sizes ranging from 50–100 nm, 100–200 nm, and 200–400 nm. These simulated “natural” NPs were more similar to naturally occurring counterparts when compared with commercial NPs. These new standard NPs, which should be scalable for large-scale use, will improve the accuracy, reliability, and translatability of toxicological studies of NPs.

## 1. Introduction

Plastic pollution has become a primary global environmental concern, with annual production exceeding 8.3 billion metric tons and increasing [1]. Although natural plastic degrades slowly, it eventually breaks down into micro- or nano-scale particles under natural conditions such as ultraviolet (UV) radiation, weathering, photodegradation, thermal degradation, mechanical degradation, and biotic degradation, after which it accumulates in the environment [2,3,4,5,6,7]. Plastic fragments have now been detected in environments as remote as Mount Qomolangma to Antarctica at diameters between 50 nm and 200 μm, highlighting their potential widespread environmental impact [8,9].

Recognizing the controversy in defining nanoplastics (NPs) by size, with <100 nm used by convention in the fields of nanomaterials and nanotechnology [9,10], here we follow previous studies and refer to any particle between 1 and 100 nm as an NP [11]. Commercially available NPs are mainly used for detection or toxicology research [12,13,14,15,16,17,18,19,20,21], but these have yet to be directly compared to naturally derived NPs [16,22,23,24,25,26,27,28,29,30,31]. Whether commercial NPs are suitable surrogates for natural NPs in these studies remains unclear. In analytical chemistry, a standard substance is usually selected such that its physicochemical properties are as similar as possible to the target of interest. Concerning their morphology, commercial NPs are different from natural NPs in size, shape, surface composition, and aggregation behavior. Specifically, commercial NPs are relatively uniform in particle size, appearance, and surface composition and show excellent mono-dispersion in ultra-pure water [23,28,32]. In contrast, naturally derived NPs are uneven in size and shape and have a complex surface composition with various polymerization forms [33]. Commercial NPs are synthesized by lotion of plastic monomer, while naturally occurring NPs are formed by natural aging (such as acid-base attack, biological digestion, and UV irradiation, etc.) [23,26]. The irregular shape of natural NPs (spherical, rod-shaped, and even lamellar) can lead to unpredictable effects, experimental error, and toxicity when replaced with uniformly spherical commercial NPs [13,23]. Many nanoparticles show biological toxicity (such as cytotoxicity, etc.) at different concentrations, and NPs are no exception [34]. Indeed, there is evidence that irregular NPs derived under natural conditions may be more toxic to organisms than uniformly spherical NPs [24,25,28,30], necessitating an authentic model NP for use in the laboratory setting.

The existing procedures for detecting NPs still have various limitations, such as mass spectrometry, which requires ionization of NPs and can damage their original state. Spectral analysis cannot detect essential parameters such as the morphology and size of NPs. Other commonly used characterization techniques, such as X-ray diffraction (XRD) and X-ray photoelectron spectroscopy (XPS), require a sufficient amount of sample to be tested to achieve a successful analysis [35,36,37]. These programs pose difficulties in characterizing natural/or simulated “natural” NPs, as these NPs are challenging to obtain and satisfy the requirements of characterizable measurements. Meanwhile, the report on NPs primarily uses the commercial NPs synthesized by lotion polymerization as the standard. However, in the natural environment, the vast majority of NPs are produced by crushing plastic products, significantly different from the formation process of commercial NPs [23]. Given that commercial NPs are significantly different from natural NPs, limiting their application as an experimental standard. Therefore, the procedure of scanning electron microscope (SEM)-particle size distribution analyzer (PSDA)-surface-enhanced Raman scattering (SERS) was established to detect NPs. The NPs of PP under simulated natural conditions were prepared by optimizing the preparation conditions, and it can be used as a standard for NPs closer to nature production and applied in research related to NPs. Our NPs will be helpful as an experimental standard to improve the quality of detection or toxicological research related to NPs.

## 2. Results and Discussion

### 2.1. Sample Preparation and Electron Microscopy

**Figure 1:**
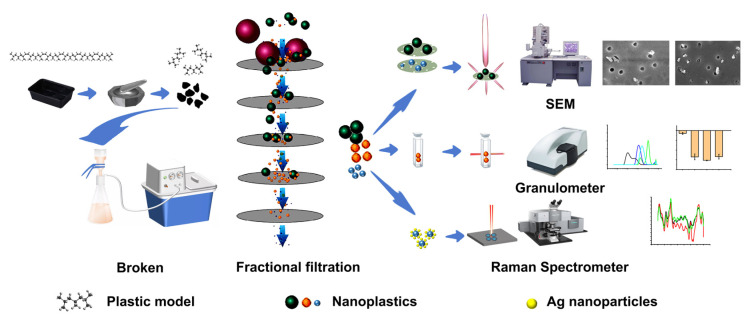
Schematic of the experiments performed in this study.

Research on the interaction between NPs and biological systems is mainly focused on commercial polystyrene. Therefore, commercial-grade polystyrene is often selected as the standard for NPs due to its broad representativeness. However, the NPs present in food consumer goods are complex and diverse. Plastic packaging materials for diet, such as polypropylene (PP), can directly contact foodstuffs and threaten human health after heating. Therefore, the PP plastic packaging materials for food have been used to prepare simulated “natural” NPs in this study (Figure 1). After acid and alkali treatment, the packaging materials are thoroughly crushed (such as shearing and grinding) and then subjected to graded filtration to obtain NPs (refer to Section 3.4 for detailed steps). Multiple chemical analysis techniques (such as SEM-PSDA-SERS) are used to characterize and analyze the obtained nanoplastics.

**Figure 2:**
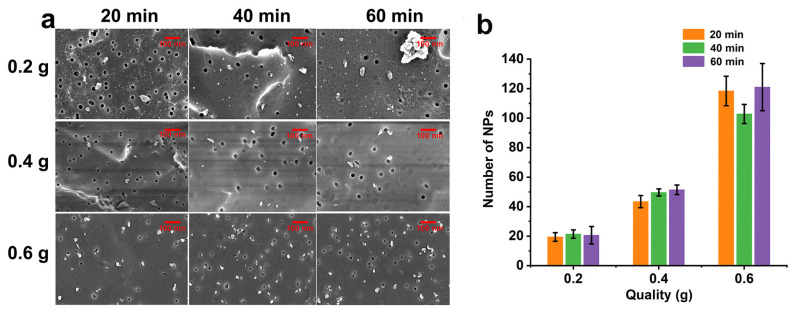
Optimization of the preparation of NPs. (**a**) Grinding time and sample quality. (**b**) The number of NPs produced under different conditions.

During preparation, since impurities produced by a glass mortar might affect the quality of NPs, we chose an agate mortar, which is more robust (Figure S1). Over the first 20 min of grinding, the size of PP plastic fragments gradually reduced. Figure 2a shows our NPs, which had a particle size of 50–100 nm as measured by scanning electron microscopy (SEM). Optimal grinding was achieved at 20 min (Figure S2), after which the density of NPs did not increase significantly with time (Figure 2). Improving the richness of NPs effectively is difficult when the grinding quality remains unchanged and the grinding time is extended. In the grinding process, the number of particles may be underestimated because the area of the particles and other factors (volume, etc.) are difficult to count. It is found that the prepared NPs have extremely irregular shape and inconsistent area by SEM, so it is hard to accurately calculate the area of the NPs by algorithms. According to Figure 2a,b, the key to effectively improving the abundance of NPs is to increase the grinding quality of the plastic, not the grinding time. It is speculated that an increase in the quality of plastic grinding is more conducive to the formation of effective collisions, thereby improving the richness of NPs.

Particle density increased according to the mass of the plastic sample, regardless of grinding time (Figure 2). Figure 2b shows the quantity of NPs in five random SEM fields. After filtration losses and grinding 0.6 g of plastic for 20 min, at least 1.24 × 10^10^ NPs were obtained according to the following calculation: N = (effective membrane filtration area)/(actual observation area of SEM) × count average, where the effective membrane filtration area was 1.73 × 10^−3^ m^2^, the actual observation area of the SEM was 1.2 × 10^−11^ m^2^, and the count average was 112. Due to filtration losses and experimental error, SEM counts can only be considered crude, and the actual quantity of NPs in the sample must have exceeded the amount observed by SEM.

Transmission electron microscopy (TEM) was used to characterize NP morphology. For this analysis, NPs were randomly adsorbed on copper foil (20 μm) for TEM visualization. To achieve this, strongly adsorbing gold nanoparticles (approximately 20 nm) were mixed with simulated “natural” NPs to promote the adsorption of NPs on the copper foil. After optimizing the grinding conditions, NPs with broad particle size distributions and irregular shapes were obtained.

### 2.2. Characterization and Analysis of Prepared NPs

The prepared NPs samples could not satisfy the minimum amount of partly chemical characterization methods (such as XRD and XPS, etc.). However, the sample size to be tested is less than 400 nm and non-conductive, which will cause energy dispersive spectroscopy electron beam breakdown and cannot be accurately characterized. In addition, this study aims to prepare NPs under simulated natural conditions, focusing on whether the particle size reaches the nanoscale. Therefore, after comprehensive consideration, the SEM-PSDA-SERS procedure was used as a characterization tool for NPs.

**Figure 3:**
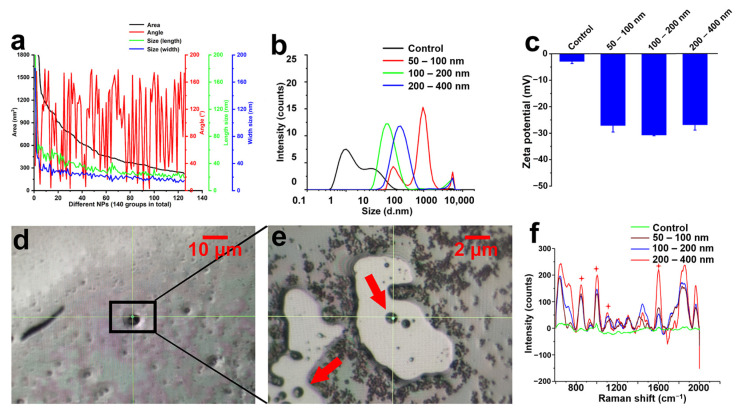
Characterizations of NPs under simulated natural conditions. (**a**) Dimensions of the NPs. (**b**) Particle size distribution of NPs using different filtration membranes. (**c**) Zeta potential of NPs of different particle sizes. (**d**,**e**) Raman spectrum scanning site (the mixture of NPs and colloidal silver is marked with a black box; the red arrow points to a mixture of colloidal silver and NPs). (**f**) Raman spectra of simulated “natural” NPs of different sizes (the red stars refer to Raman characteristic peaks of PP NPs).

Figure 3a shows the area, symmetry, length, and width of our prepared NPs under simulated natural conditions, while Figure 3b shows the size distribution as measured by PSDA based on optical principles. The most common diameter of NPs obtained after filtration using 200 and 400 nm-pore diameter polycarbonate films was 260.0 ± 5.0 nm, while with pore diameters of 100 and 200 nm, the size was 119 ± 3.0 nm. According to Figure 3b, the particle size of simulated “natural” NPs filtered by the 50–100 nm polycarbonate membrane mainly was 960 ± 5 nm, which is speculated to be due to the easy aggregation of nanoscale plastic particles in the sample [38,39]. It was speculated that it was caused by the accumulation phenomenon of NPs prepared under simulated natural conditions. However, the NPs obtained using films with pore diameters of 50–100 nm were aggregated, and the NPs with particle sizes of 164 nm and 965 nm accounted for 23.4% and 78.2%, respectively. The boundary of particle dispersion stability in the aqueous phase is generally considered at a Zeta potential of +30 mV or −30 mV. Concerning Zeta potential, NPs of all three sizes approached −30 mV (Figure 3c), indicating that all three sets of NPs of different particle sizes were relatively stable in ultra-pure water, as the Zeta potential of the measured NPs is close to −30 mV.

Traditional Raman is reported to be most suitable for microplastics larger than 10 μm in actual environmental samples [21]. Surface-enhanced Raman spectroscopy (SERS) has great potential for measuring particles that are smaller than the diffraction limit of conventional Raman spectroscopy [40,41]. Raman signal can be enhanced significantly in the very small spatial region (<10 nm) that is activated by assembly of metallic nanoparticles. Therefore, the purpose of mixing colloidal silver and NPs for SERS measurement in this study is to enhance the Raman signal of NPs by utilizing the strong surface plasmon resonance excited by silver nanoparticles. SERS was used to further characterize the NPs. Silver nanoparticles were adsorbed on the surface of NPs in ultra-pure water. As the condensation core, NPs can attract the surrounding unabsorbed silver particles to form spots of about 1 µm (Figure 3d,e) when the silicon wafer is dry. Raman characteristic PP peaks were observed at 841 cm^−1^, 971 cm^−1^, 1149 cm^−1^, and 1451 cm^−1^ for NPs of three particle sizes (Figure 3f). The results show that the composition of NPs under simulated natural conditions is PP, which indicates that the NPs simulated by natural conditions have been successfully prepared in this study. Therefore, these data show that we successfully prepared NPs from PP packaging material.

### 2.3. Comparison of Commercial NPs, Simulated “Natural” NPs, and Naturally Derived NPs

The simulated “natural” NPs of particle size 50–100 nm, commercial NPs (50 nm), and NPs of particle size 50–100 nm derived under natural conditions were compared in a follow-up experiment.

**Figure 4:**
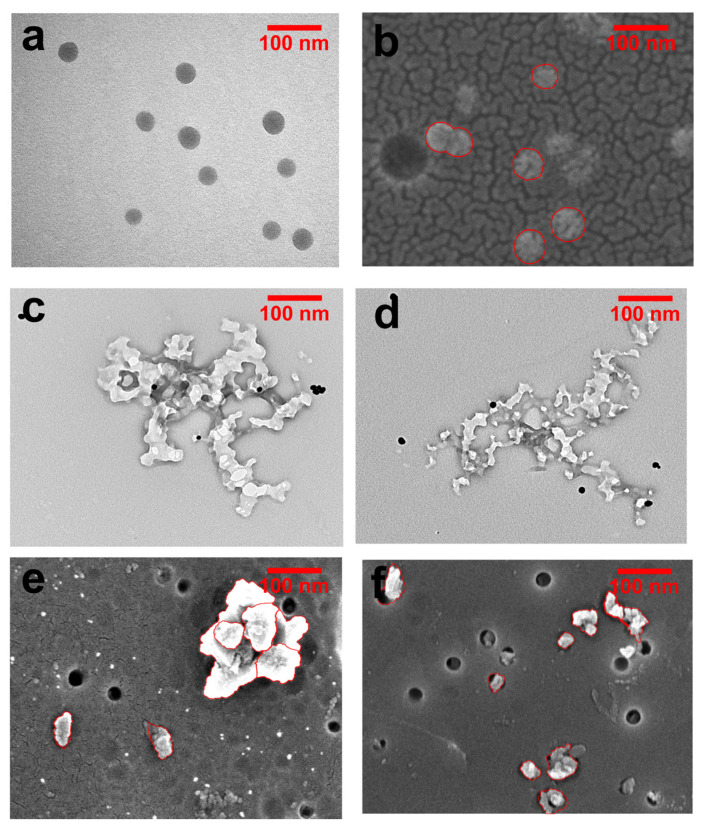
Representative images of three types of NPs. (**a**) Monodisperse commercial NPs with a 50 nm particle size under TEM. (**b**) Monodisperse commercial NPs with a 50 nm particle size under SEM. (**c**) Simulated “natural” NPs with 50–100 nm particle size under TEM. (**d**) Naturally derived NPs with a 50–100 nm particle size detected by TEM. (**e**) Simulated “natural” NPs with a 50–100 nm particle size under SEM. (**f**) Naturally derived NPs with a 50–100 nm particle size under SEM.

NP shapes were analyzed by SEM and TEM (Figure 4). The distribution of commercial NPs was shown to be uniform. It is speculated that most commercial NPs are produced through lotion polymerization, and the surfactant on their surfaces can reduce the surface energy, thus generating steric hindrance and improving the stability of particles. By both TEM and SEM, our synthetic NPs and naturally derived NPs were irregular and showed signs of aggregation.

**Figure 5:**
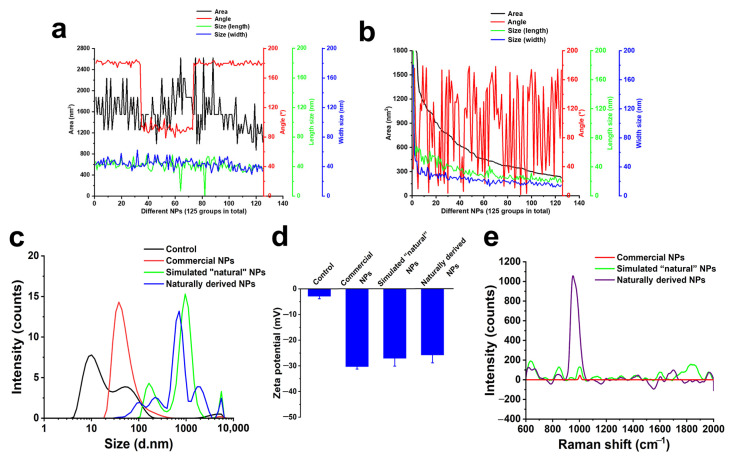
Comparison of the different types of 50–100 nm NPs. (**a**,**b**) Size parameters of the two types of NPs ((**a**): commercial NPs; (**b**): naturally derived NPs). (**c**) Size distribution of the three types of NPs (Control: ultra-pure water). (**d**) Zeta potential of the three types of NPs (Control: ultra-pure water). (**e**) Raman spectra of the three types of NPs.

Based on our characterization analyses (Figure 3a and Figure 5b), compared with commercial NPs (Figure 5a), simulated “natural” NPs (Figure 3a) were more similar to natural NPs (Figure 5b). Non “natural” NPs were prepared by simulating the formation pathway of NPs under natural conditions.

The particle size distribution of commercial NPs was uniform without any aggregation, while the distribution of simulated NPs was similar to naturally derived NPs, with particles larger than the commercial forms (Figure 5c). Zeta potential analysis suggested no significant difference between naturally derived NPs and simulated “natural” NPs compared to commercial NPs (Figure 5d).

Raman information of simulated “natural” NPs can be analyzed using SERS procedure [21]. The simulated “natural” NPs (951.368 cm^−1^) and naturally derived NPs (1001.7 cm^−1^) exhibited similar characteristic Raman peaks of their original polypropylene materials (1003.06 cm^−1^), and the results indicated that the composition of these NPs has not changed, only reached nanoscale dimensions in size (Figure 5e).

### 2.4. Eccentricity Statistics

**Figure 6:**
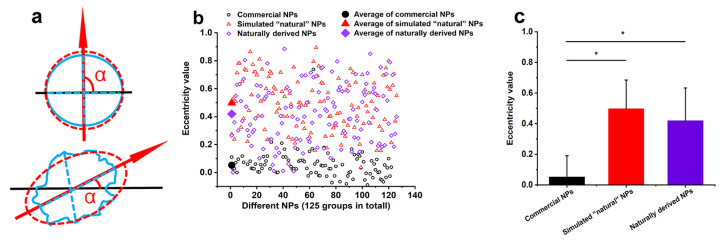
(**a**) Schematic of the assessment of NP shape (black line: axis of symmetry; blue line: the actual shape of the NPs; red line: ellipses converted by NPs). (**b**) The eccentricity of three different types of NPs (The abscissa represents the number of NPs; The hollow shapes represent the eccentricity of a single NP, while the solid shapes represent the average eccentricities of all NPs in each group). (**c**) The average eccentricities of commercial NPs, synthetic “natural” NPs, and naturally derived NPs. *: *p* < 0.05.

To assess the eccentricity of NPs, we next considered how close the particle shape was to a perfect circle or ellipse (Figure 6a). According to this approach, an eccentricity value of 0 denoted a circle, while a value between 0 and 1 denoted an elliptical shape.

Simulated “natural” NPs and those produced under natural conditions had eccentricity values between 0 and 1 (Figure 6b), with average eccentricities of 0.497 and 0.420, respectively, and no significant difference between the two groups. The data proves that the centrifugal rates of simulated “natural” NPs and naturally derived NPs are relatively similar, closer to ellipses, and have significant differences from commercial NPs that are close to perfect circles (Figure 6c).

## 3. Materials and Methods

### 3.1. Materials and Reagents

A takeout PP tableware box was purchased from RT-MART (Taiwan, China). Spherical polypropylene NPs (commercial NPs) were supplied by Jiangsu Zhichuan Technology Co., Ltd. (Yixing, China), and polycarbonate filter membranes were sourced from Whatman (Maidstone, UK). Sodium hydroxide (NaOH), hydrochloric acid (HCl), magnesium sulfate (MgSO_4_), silver nitrate (AgNO_3_), hydroxylamine hydrochloride (HONH_3_Cl), chloroauric acid (AuCl_4_H), and trisodium citrate (C_6_H_5_Na_3_O_7_) were purchased from Sinopharm Chemical Reagent Co., Ltd. (Shanghai, China).

### 3.2. Instruments

Sampled NPs were scanned by TEM (HT-7800, Hitachi, Tokyo, Japan) at an acceleration voltage of 80 kV. SEM (GeminiUltra-55; Carl Zeiss AG, Oberkochen, Germany) was used to observe the surface and section morphology of NPs. The particle size distribution of sampled NPs was determined using a laser particle size analyzer (ZEN-3600, Malvern Panalytical, Malvern, UK). Ultra-pure water was obtained from an ultra-pure water meter (Pure Force RO-300, Heal Force, Shanghai, China). SERS spectra were captured using confocal Raman microscopy (Lab RAM HR Evolution, Horiba, Kyoto, Japan), with a He-Ne (633 nm) laser as the excitation source.

### 3.3. Preparation of Colloidal Silver and Gold

Colloidal silver was prepared using the method proposed by Leopold et al. [42]. AgNO_3_ solution (90 mL, 1 mM) was mixed with a solution containing HONH_3_Cl (10 mL, 1.67 mM) and NaOH (3.33 mM) with vigorous stirring. Then, the mixture was stirred at room temperature for another 10 min. Colloidal gold was prepared using the method developed by Jia et al. [43]. With the C_6_H_5_Na_3_O_7_ (2.0 mL, 0.039 mM) added to the boiling HAuCl_4_ (100 mL 0.03 mM) solution, boiling was maintained, and stirring continued until the solution changed from grayish-white to wine-red. At this point, heating was stopped, but stirring continued until the mixture cooled to room temperature.

### 3.4. Preparation of NPs

(1) Preparation of simulated “natural” NPs

Simulated “natural” NPs are a type of NP produced in a laboratory that simulates natural conditions (such as microorganisms, acid-base erosion, UV exposure, etc.). Simulated “natural” NPs were prepared in the laboratory, and mortar selection, grinding quality, and grinding time were optimized. PP boxes were immersed in ultra-pure water (pH = 7.0), 0.25% porcine trypsin, HCl (pH = 4.0) and NaOH (pH = 10.0) for 24 h at each step, respectively (the box needs to be cleaned five times with ultra-pure water at the end of each step) [19,44,45]. The soaking solution was changed every 6 h. The use of enzymes to treat plastics is to simulate the process of biological digestion. In addition, on the one hand, the use of acid and alkali can simulate the impact of acid and alkali erosion processes on plastic products in the environment. The presence of acidic or alkaline substances in aqueous solutions would tend to corrode the surface of plastics, thus promoting the damage process of micro(nano)plastic particles over long-term contact [46]. On the other hand, acid and alkali treatment of plastic surfaces can effectively remove organic matter polluted by the environment. Then, the processed PP boxes were immersed in ultra-pure water for 24 h (the ultra-pure water was changed every 6 h) and dried at room temperature, then the dried PP boxes were exposed to UV light for 24 h. Afterward, PP boxes were split into small pieces of about 1 mm^2^ using scissors, and 0.6 g of PP pieces were ground in an agate mortar for 20 min and then rinsed with ultra-pure water.

Polycarbonate membranes with an aperture of 400 nm, 200 nm, 100 nm, and 50 nm and a diameter of 50 mm were used for fractionation filtration, with the aperture membranes of 200 nm, 100 nm, and 50 nm retained. Synthetic simulated “natural” NPs with a particle size of 200–400 nm/100–200 nm/50–100 nm were obtained. The central part of the polycarbonate membranes (25 mm^2^) was sprayed with gold for SEM, while the remainder was placed in a beaker filled with 5 mL ultra-pure water. After 30 min of ultrasound treatment, NPs were added to ultra-pure water. Eventually, ultra-pure water-dispersed samples of simulated “natural” NPs of different particle sizes were obtained.

(2) Preparation of naturally derived NPs

PP boxes were bathed in boiling water (100 °C) and cooled to room temperature to mimic the process of natural aging of plastic materials (such as heat treatment, weathering, etc.) and obtained naturally derived NP samples [47]. NPs of different particle sizes were obtained after fractional filtration, and these three varieties of NPs of different particle sizes were obtained on both membranes and as ultra-pure water-dispersed samples.

### 3.5. Characterization of NPs

The NPs sample suspension was mixed with colloidal gold solution (*v*/*v* = 1:1), and the mixture was continuously and slowly shaken for about 30 min to cause electrostatic repulsion between NPs and gold nanoparticles (AuNPs) [48,49]. Then the mixture was covered with copper foil and observed by TEM. The samples were observed on polycarbonate membranes by SEM (3 kV) to characterize their morphology and richness. In addition, the gold-sprayed NPs samples were observed by SEM. The obtained SEM images were analyzed by Image Pro Plus 6.0, including particle number, size, shape, and other parameters. Ultra-pure water-dispersed samples were observed by TEM (8 kV) and detected with a particle size analyzer, thus further verifying their morphology and size. The particle size distribution and Zeta potential of NPs were analyzed using the particle size analyzer. With 20 μL of liquid sample and 1 mL of colloidal silver solution mixed evenly (vortex for 1 min approximately), 10 μL of magnesium sulfate solution was added to the sample (1 mol/L) and mixed evenly. Then, 5 μL of the mixture was removed, placed on clean silicon wafers, and dried (5 min, 60 °C) for scanning by confocal Raman microscopy. The SERS technique was used to test Raman signals from NPs, and the surface was enhanced with silver nanoparticles [23].

### 3.6. Data Analysis

Photoshop CS6 (Adobe, San Jose, CA, USA) was used to process images. Image Pro Plus 6.0 (Media Cybernetics, Rockville, MD, USA) was used to analyze SEM images. Origin 2021 (OriginLab, Northampton, MA, USA) was used to analyze particle size, Zeta potential, and Raman spectra. SPSS software (IBM Statistics, Chicago, IL, USA) was used to assess significance with a *p*-value threshold of ≤0.05.

## 4. Conclusions

There are significant differences in the preparation principles between traditional commercialized NPs and naturally derived NPs, as well as in the biological toxicity or potential biological function of commercial NPs and naturally occurring NPs. To ensure the accuracy of scientific experiments, the NP materials used in scientific research should be consistent with those with high environment content. NPs produced under natural simulation conditions with a particle size distribution range of 50–100/100–200/200–400 nm were obtained using a graded filtration method. After characterization using the procedure of SEM-PSDA-SERS, irregular granular NPs with certain aggregation phenomena in the range of corresponding separation levels were obtained. Our method produced NPs with similar characteristics to naturally derived NPs (but not commercial NPs), thereby overcoming the problem that commercial NPs need to simulate naturally derived NPs accurately. In the future, manual grinding can be replaced by mechanical procedures such as ball mills and other blending method for mass production after a series of optimizations and measurements. Meanwhile, biochemical degradation can also be an essential source of naturally derived NPs in the future, and its degradation particles can be further obtained by simulating the biochemical degradation of plastic materials. Subsequently, in vivo (plant or animal) toxicological tests on the naturally generated NPs will provide valuable data for further risk assessment. Further, high-quality NP standards are expected to facilitate future research on NPs and improve their accuracy, reliability, and translatability in real-world settings.

## Data Availability

Not applicable.

## Supplementary Materials

The following supporting information can be downloaded at https://www.mdpi.com/article/10.3390/molecules28217254/s1, Figure S1: Schematic diagram of mortar selection. (a,b) Particles produced in the glass mortar during grinding. (c,d) Particles produced in an agate mortar during grinding; Figure S2: Image of the change occurring to plastic particles in the grinding process. (a–d) The corresponding grinding time is 0 min, 10 min, 20 min and 30 min, respectively; Figure S3: TEM characterization of gold and silver nanoparticles. (a) Gold nanoparticles with a particle size of 40 nm. (b) Silver nanoparticles with a particle size of 20 nm; Figure S4: Diagram of image processing by Image Pro Plus 6.0 software processes image. (a,d) Scanning electron microscope characteristic diagram of simulated “natural” NPs and naturally derived NPs. The software is used to select (b,e) and measure (c,f) the parameters of NPs; Figure S5: Observation of NPs by TEM. (a) Preparation of NPs standards under simulated natural conditions by TEM. (b) Preparation of NPs standard under simulated natural conditions plus colloidal gold under TEM.
