# Supplementary material for: Generation of Simulated “Natural” Nanoplastics from Polypropylene Food Packaging as the Experimental Standard
Source: Molecules. 2023 Oct 25;28(21):7254. doi: 10.3390/molecules28217254 (PMC10648906; doi:10.3390/molecules28217254)
Supplement: Supplementary file 1 [file molecules-28-07254-s001.zip › molecules-2673959-supplementary.pdf]

Supporting Information

Generation of Simulated "Natural" Nanoplastics from Polypropylene Food Packaging as the Experimental Standard

Zhongtang Wang, Ying Wang, Xin Lu, Hongyan Zhang and Zhenzhen Jia*

Shandong Provincial Key Laboratory of Animal Resistance Biology, Key Laboratory of Food Nutrition and Safety of Shandong Normal University, College of Life Sciences, Shandong Normal University, Jinan, China

*Correspondence: jiazhenzhen@sdnu.edu.cn (Zhenzhen Jia)

Figure S1, **S2**, **S3**, **S4 and S5** show the schematic diagram of how the mortar is selected, the schematic diagram of how the basic grinding time is determined, the diagram of transmission electron microscope (TEM) characterization for gold and silver nanoparticles, and the schematic diagram of particle parameters as measured using the Image Pro Plus 6.0 software, respectively. And the observation of NPs by TEM.

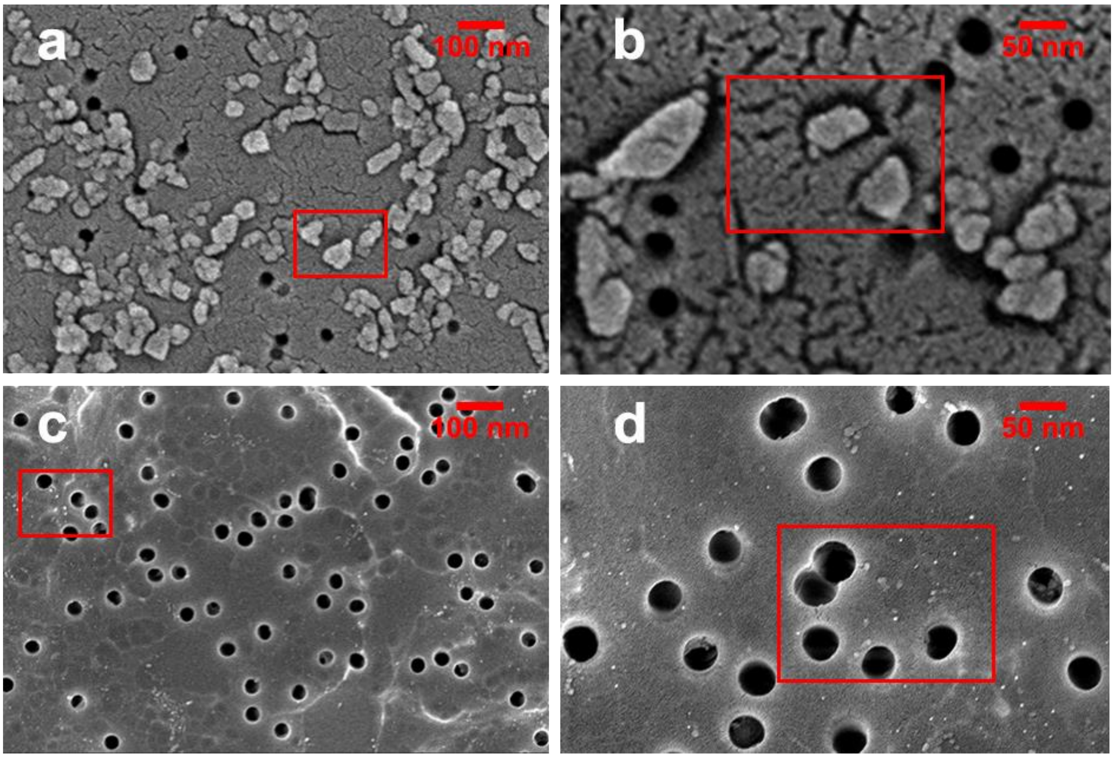

Figure S1. Schematic diagram of mortar selection. a, b) Particles produced in the glass mortar during grinding. c, d) Particles produced in an agate mortar during grinding.

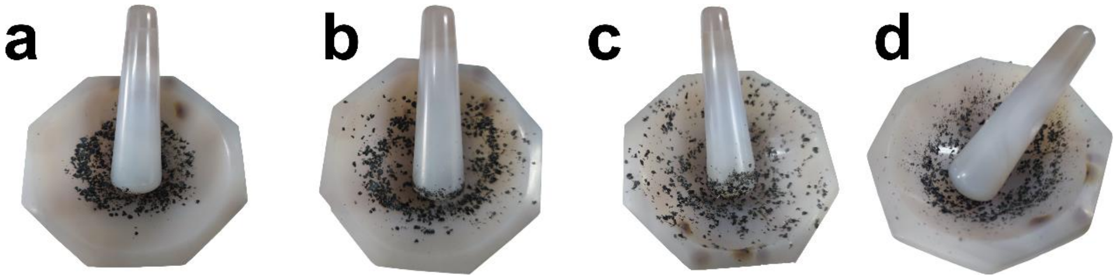

Figure S2. Image of the change occurring to plastic particles in the grinding process. (a~d) The corresponding grinding time is 0 min, 10 min, 20 min and 30 min, respectively.

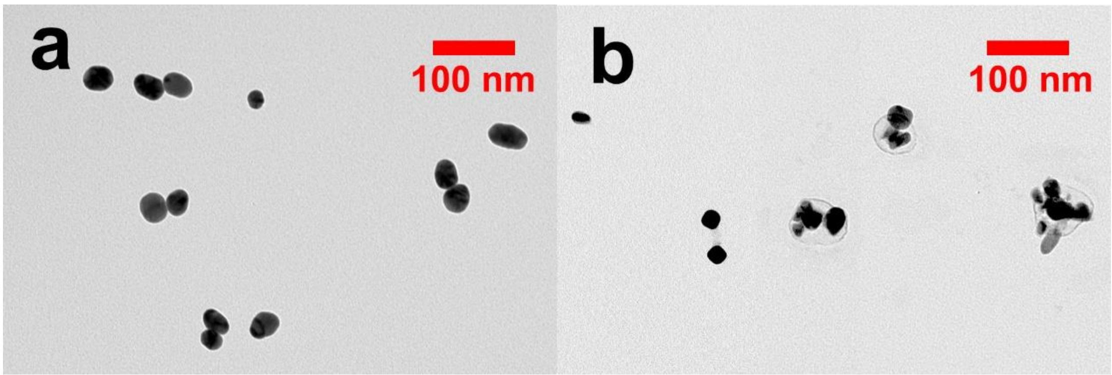

Figure S3. TEM characterization of gold and silver nanoparticles. a) Gold nanoparticles with a particle size less than 100 nm. b) Silver nanoparticles with a particle size less than 100 nm.

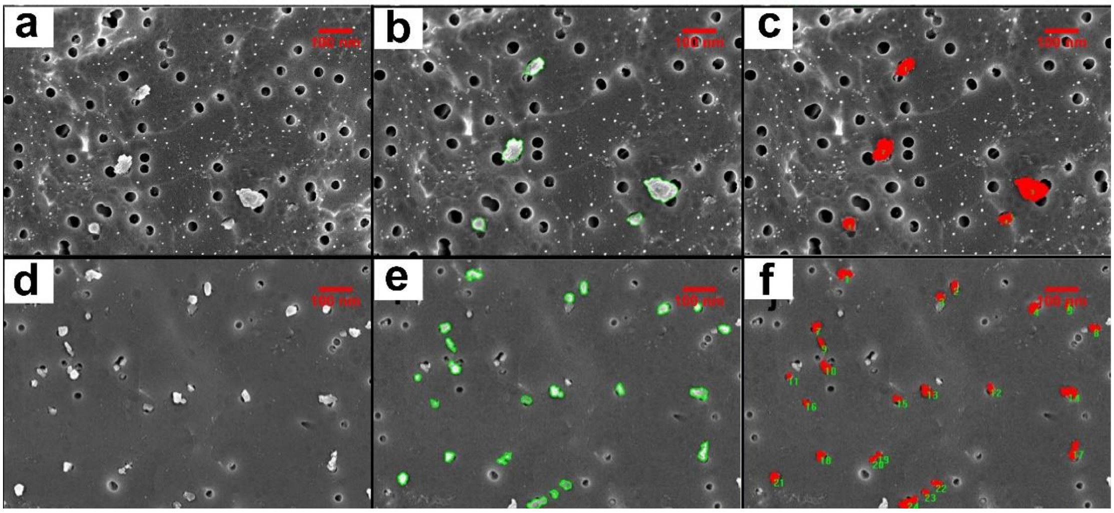

Figure S4. Diagram of image processing by Image Pro Plus 6.0 software processes image. a, d) Scanning electron microscope characteristic diagram of self-made and naturally-derived NPs. The software is used to select (b, e) and measure (c, f) the parameters of NPs.

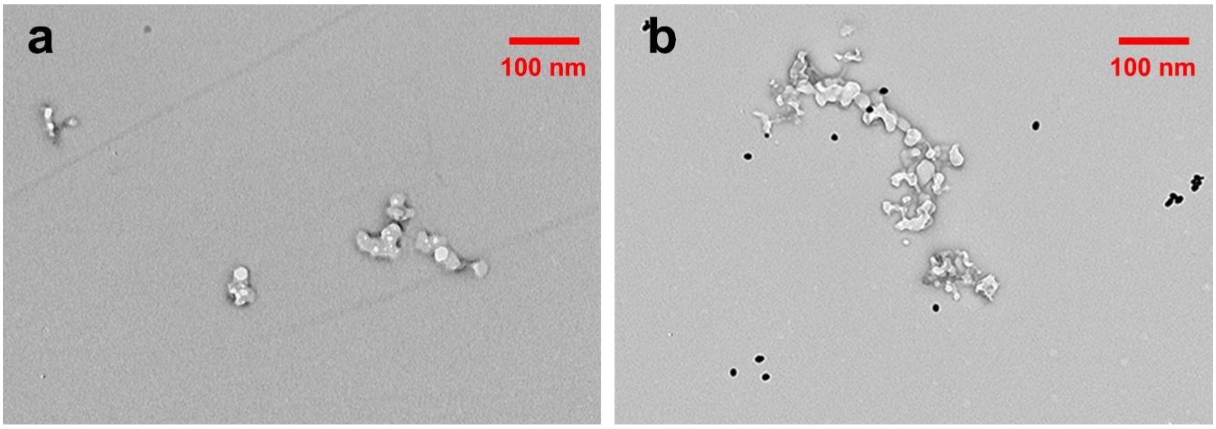

Figure S5. Observation of NPs by TEM. a) Preparation of NPs standards under simulated natural conditions by TEM. b) Preparation of NPs standard under simulated natural conditions plus colloidal gold under TEM.
